# Cessation of thumb/finger sucking habit in children using electronic habit reminder versus palatal crib: a randomized clinical pilot study

**DOI:** 10.1186/s12903-024-05310-6

**Published:** 2025-01-06

**Authors:** Tassneim Eltager, Adel El Bardissy, Fatma Abdelgawad

**Affiliations:** Paediatric Dentistry and Dental Public Health Department, Faculty of Dentistry, Cairo University, EL-Saraya Street, Cairo, Egypt

**Keywords:** Thumb/finger sucking, Electronic habit reminder, Palatal crib, Children

## Abstract

**Background:**

There are different intraoral appliances for cessation of thumb/finger sucking habit, but they have many disadvantages and to overcome it, extra oral appliances with colourful and attractive shape were developed. Electronic habit reminder in the form of wristwatch with alarming sound was assessed in cessation of thumb/finger sucking habit in children versus palatal crib after 6 and 9 months.

**Methods:**

This study is a randomized clinical pilot study, with allocation ratio 1:1 parallel group. Recruitment was at the diagnostic clinic, Paediatric Dentistry and Dental Public health Department, Faculty of Dentistry, Cairo University. Blinding was not feasible except for the statistician.

Twenty-two children were included with age range (6–14), erupted upper first permanent molar and with thumb/finger sucking habit that resulted in open bite. After random allocation of participants into two groups: intervention group (electronic habit reminder) and control group (palatal crib), impressions were performed for fabrication of the appliances in both groups. Follow up was performed at 2 weeks, 1, 3, 6 and 9 months. Primary outcome was assessing cessation of thumb/finger sucking habit in children.

**Results:**

The total number of participants who were randomized and analysed was 22 (11 per group). Cessation of thumb/finger sucking habit in the intervention group was 27.3% while in the control group was 54.5% but with no statistically significant difference (*P˃*0.05). Positive feedback from the parents about the useful instructions, success, and ease of using the appliances but all with no statistically significant difference (*P˃*0.05).

**Harms:**

Regarding the palatal crib appliance, there was gingival inflammation that resolved by proper oral hygiene care. Also, interference with speech which disappeared after adaptation.

Regarding appliances breakage or dislodgment, it was repaired or replaced with another one.

**Conclusion:**

Although most of the parents and children accepted both appliances, cessation of the habit was higher in the control group than in the intervention group.

**Trial registration:**

The trial was registered on clinicaltrials.gov, ‘Trial registration number: NCT04075617 [first submitted -29/8/2019]’.

## Background

Craniofacial growth and development are affected by several actions like breathing, chewing, swallowing and sucking [1]. The habit is a continuous duplication of an act [2]. Oral habits like thumb/finger sucking, nail biting, lip chewing, tongue thrust, pacifier use and bruxism [3]. Oral habits surprisingly have benefits like lowering caries incidence. There are 2 case control studies that reported the benefit of thumb/finger sucking habit in preventing sudden infant death syndrome (SIDS) but there is not enough evidence [4, 5].

Oral habits have a great impact on the developing occlusion in young children. Intensity, duration, and frequency of these habits are directly proportional to the degree of normal occlusion upset [6]. There is a high incidence of oral habits in children with the age range 3–5 years old [7, 8]. In a study conducted in India, oral habits were recognized in 36% of the participant children. With prevalence being12.8% in both thumb sucking and bruxism [8]. Children use thumb/finger sucking as a tool to ease stress or pressure and help in sleeping. In comparison to the past decades, these habits are increasing due to more stressful conditions and environments [9].

During the development of facial structures, bone, and teeth, they are all under balanced forces to reach normal position and occlusion. So, when a habit such as thumb/finger sucking is persistent during the growth of the child for several hours per day, it will disturb these forces causing malocclusion [10]. The effects on primary dentition are commonly seen as labial tipping of anterior teeth and sometimes interference with the path of eruption [11]. With prolonged thumb/finger sucking, there will be posterior equilibration of the downward movement of the mandible by 1 mm that results in 2 mm of anterior bite opening [10].

Management of thumb/finger sucking habit is different according to the child’s age, in primary dentition no treatment will be applied, and any dental changes will be self-corrected if the child stops the habit early. But if the habit persists in early mixed dentition, the parents should start encouraging the child to stop the habit by rewards and simple methods like applying bitter material or bandage. If the habit continues in mixed dentition, professional appliances are used with high success rates. While the effects on permanent dentition corrected only by orthodontic treatment. Early intervention should be considered as assistance and not penalizing [12, 13]. Treatment by professional methods (reminder or orthodontic therapy) have higher success rates compared to common methods applied by the parents at home [14].

Intraoral appliances commonly used quad helix and palatal crib [15]. High success of palatal crib due to ability in modifying tongue posture leading to cessation of the thumb sucking habit [16]. Children may struggle with eating and temporarily change in speaking but usually adapt to this problem within a week [13].

Extra oral approaches are well accepted by school-age children [17]. Thumb guard, RURS elbow guard and “three-alarm system: revisited” are recent extra oral appliances, designed to be worn on the finger/elbow to remind the child not to suck his/her thumb/finger [32]. Krishnappa et al., introduced electronic habit reminder in the form of wristwatch with attractive appearance to be used in cessation of thumb/finger sucking habit. It was effective in stopping the habit and the child accepted it easily with no problems reported with its use [18]. No studies were found to assess the use of electronic habit remainder to overcome the disadvantage of using palatal crib, therefore this study was conducted to compare the effect of using extra oral electronic habit remainder versus fixed palatal crib after 6 and 9 months of treatments.

## Methods

### Trial design

This study is a randomized clinical pilot study, with allocation ratio 1:1 parallel group.

Ethical approval was granted by the Research Ethics Committee (REC), Faculty of Dentistry, Cairo University and approved on 26/11/2019 with approval number 9–11–19. The study timeline was from December 2019 to February 2023. It was registered on clinicaltrials.gov with registration number: NCT04075617. It follows the CONSORT guidelines.

### Participants

The study was conducted in the postgraduate clinic, Paediatric Dentistry and Dental Public Health Department, Faculty of Dentistry, Cairo University, Egypt.

#### Operator

Master’s degree student in Paediatric Dentistry and Dental Public Health Department, Faculty of Dentistry, Cairo University, Egypt.

Recruitment was at the diagnostic clinic, Paediatric Dentistry and Dental Public health Department, Faculty of Dentistry, Cairo University, it was performed by TE. The parents were asked if their children have thumb/finger sucking habit. Clinical examination for the oral cavity and thumb/finger was performed to ensure that the children have sucking habit. All included children had no speech problems. Among the inclusion criteria, they have anterior open bite and erupted upper first permanent molar. While, the exclusion criteria was children with special health care needs, uncooperative children and parents refuse the participation of their children. The trial was discussed with the parent/guardian of each child and a written informed consent was signed while verbal assent confirmed orally from the child if more than 7 years old. The parent/guardian was asked about history of breastfeeding/bottle-feeding, mother occupation (working or not), the order of the child among his/her siblings and previous treatments used to overcome the sucking habit. Twenty two children were included with age range 6–14, in each group 11 participants in the intervention (males: 6, females: 5) while in control (males: 5, females: 6).

### Interventions

**Fig. 1:**
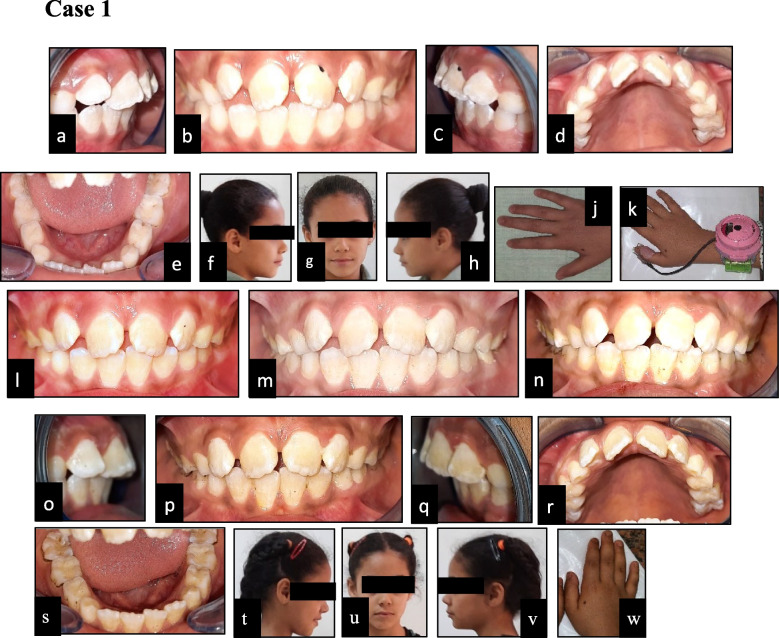
**a**-**k** Showing preoperative photos of intervention case that stopped the thumb/finger sucking habit: (**a**) right lateral intraoral view; (**b**) frontal view; (**c**) left lateral intraoral view; (**d**) maxillary occlusal view (**e**) mandibular occlusal view; (**f**) right profile view; (**g**) frontal view; (**h**) left profile view; (**j**) hand photo; (**k**) hand photo with the electronic habit reminder. **l**-**n** Showing postoperative photos: (**l**) follow up at 1 month; (**m**) follow up at 3 months; (**n**) follow up at 6 months. **o**-**w** Showing postoperative photos at 9 months: (**o**) right lateral intraoral view; (**p**) frontal view; (**q**) left lateral intraoral view; (**r**) maxillary occlusal view (**s**) mandibular occlusal view; (**t**) right profile view; (**u**) frontal view; (**v**) left profile view; (**w**) hand photo

**Fig. 2:**
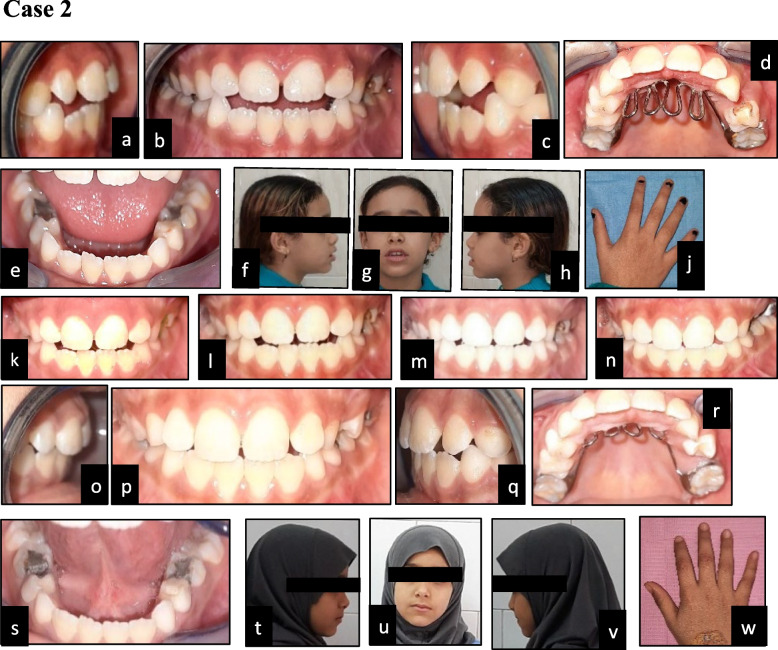
**a**-**j** Showing preoperative photos of control case that stopped the thumb/finger sucking habit: (**a**) right lateral intraoral view; (**b**) frontal view; (**c**) left lateral intraoral view; (**d**) maxillary occlusal view with palatal crib (**e**) mandibular occlusal view; (**f**) right profile view; (**g**) frontal view; (**h**) left profile view; (**j**) hand photo. **k**-**n** Showing postoperative photos: (**k**) follow up at 2 weeks; (**l**) follow up at 1 month; (**m**) follow up at 3 months; (**n**) follow up at 6 months. **o**-**w** Showing postoperative photos at 9 months: (**o**) right lateral intraoral view; (**p**) frontal view; (**q**) left lateral intraoral view; (**r**) maxillary occlusal view with palatal crib, (**s**) mandibular occlusal view; (**t**) right profile view; (**u**) frontal view; (**v**) left profile view; (**w**) hand photo

In this study, we assessed cessation of thumb/finger sucking habit using electronic habit reminder in the intervention group (shown in Fig. 1) and palatal crib in the control group (shown in Fig. 2). After diagnosis and enrolment, intraoral photos were taken for all participants with paediatric cheek retractor (Elephant cheek retractor, China) and extra oral profile photos and hand photo.

#### In intervention group

Impression for the thumb/finger used in the sucking habit was performed using putty consistency silicon impression material (Zhermack Zetaplus, Italy). This impression was poured by dental stone to formulate the first part of the appliance which fits on the thumb/finger. Then stainless-steel wires size 0.9 and 0.7 were adapted to fit the finger in the form of inverted U shape. Different sizes of the wires were used for better adaptation on the finger and to be harder for the child to deform. Stainless-steel wires were connected to the wristwatch with electronic wires which were covered with heat shrink material for stabilization and protection from being pulled apart by the child.

**Fig. 3:**
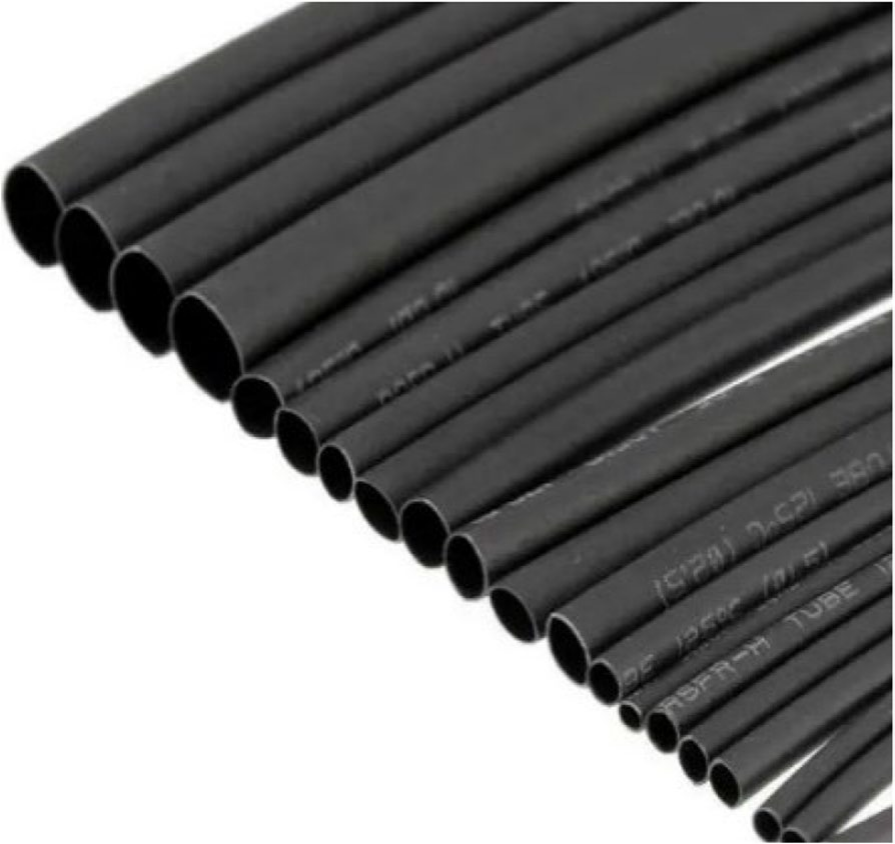
A photograph showing heat shrink material
- The wristwatch used was a regular child watch with some modifications (shown in Fig. 4). It had the advantage of producing an alarming sound which was turned on by a button. This button was removed and the electronic wires from it were soldered to the wires from the first part. So that, the child places the thumb/finger in the mouth, alarm sound is produced when the stainless-steel wires touch each other by pressure from sucking the thumb/finger and stops when the child removes the thumb/finger.

- The acrylic material (Acrostone acrylic material cold cure, Egypt) was used in the form of a ring to enclose the stainless-steel wires and the electronic wires with the covering heat shrink (shown in Fig. 3). While the other end of the covered electronic wires was inserted in an opening present on the side of the wristwatch.

**Fig. 4:**
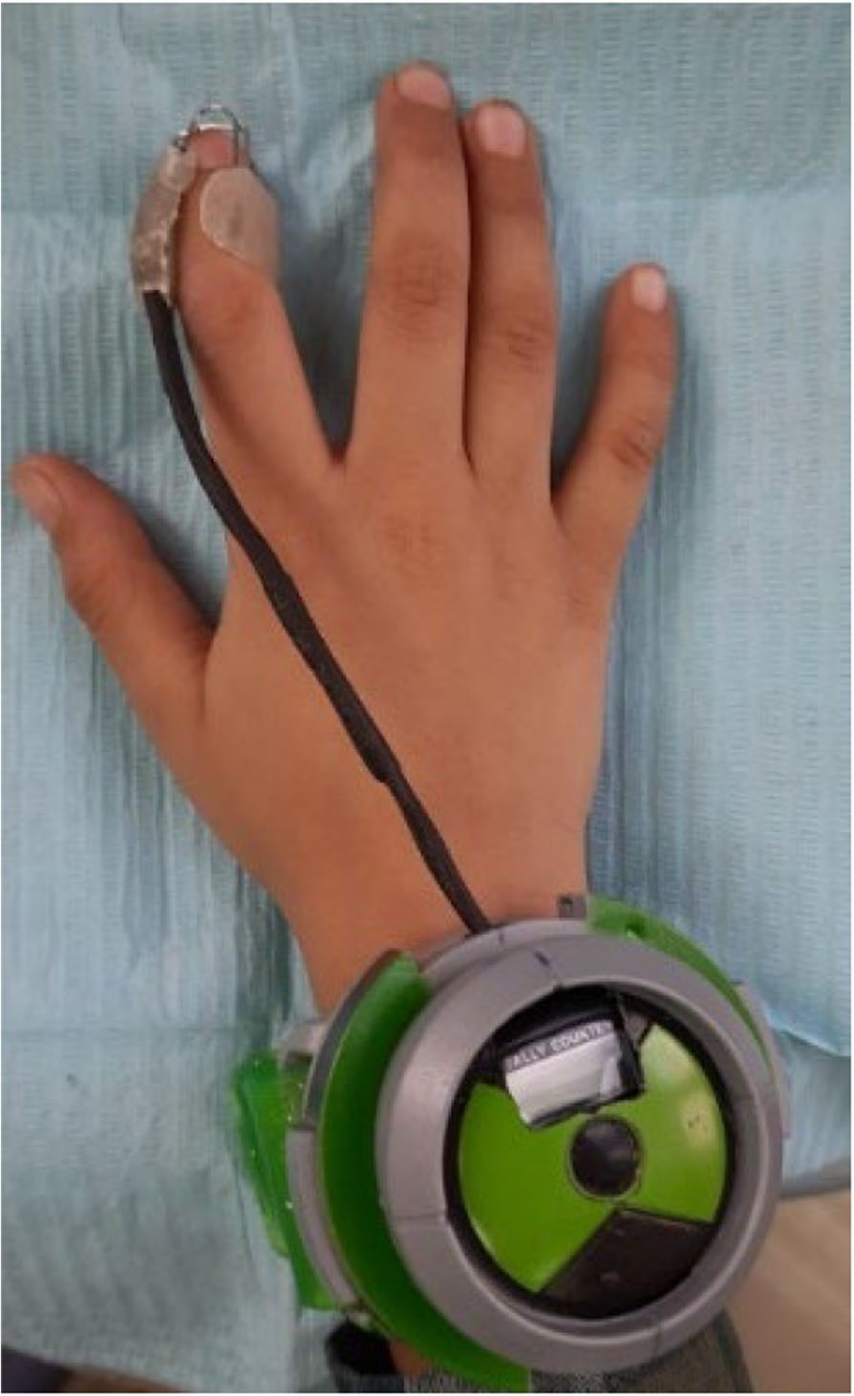
A photograph showing electronic wristwatch with the attached finger part
- Additional part was inserted inside the wristwatch in the form of electric counter to record number of times the child attempts to suck their thumb/finger. It was soldered to electronic wires which was connected to the stainless-steel wires and the acrylic part.
- After a week, the parent/guardian and the child were called to receive the appliance, instructions for wearing it all the time except while eating and how to record in the booklet (follow up chart) day by day.

#### In control group

Impression for the upper arch and the lower arch were performed using size 1 dentulous tray (Aluminum impression tray, Egypt) with alginate material (Cavex CA37 normal set, Haarlem, The Netherlands). The impression was used for fabrication of the palatal crib. After a week, the parent and the child were called to receive the appliance. After checking the fitting of the appliance and the occlusion. It was cemented by glass ionomer cement (Nova Glass L, R&D series, Turkey).

#### Follow up

Oral hygiene instructions were provided to the parents/guardian and the child to regularly brush their children’s teeth to avoid inflammation and food stagnation especially with the control group after each meal. The parents were advised to make a phone call to the operator if there is any problem. The original timeline for treatment was 6 months but it was extended to 9 months for actual effects to be recorded. Follow up was performed on 2 weeks, 1, 3, 6 and 9 months. To avoid relapse of the habit, parents/guardians were instructed to have their children wear the appliance continuously for another 6 months in case of the intervention group. While, the control group, we removed it at the clinic after the additional 6 months.

## Outcomes

**Table:** 

**Prioritization of Outcome **	**Outcome**	**Method of Measurement **	**Unit of Measurement**
Primary outcome	Cessation of thumb sucking habit	Asking the parent/guardian [18]	Binary (yes or no)
Secondary outcome	1- Child acceptance	Asking the child [18]	Binary (yes or no)
	2- Parental acceptance	Questionnaire [19]	Scale from 1:5
	3- Time for habit cessation	Booklet (follow up chart) [20]	Days/ months

### Sample size

Using the electronic habit reminder for cessation of thumb/finger sucking habit was never used before except in one case study [18]. So, this study is considered a pilot study. The suggested sample size was 22 children according to Hertzog who suggested pilot study estimation [21].

## Randomization and allocation

Sequence generation was performed on the random.org website to produce list of random numbers that were divided into two groups (intervention and control). This list of numbers was with FA. Allocation concealment was performed by making a phone call to FA to know which group the participant was allocated to. Implementation was performed by providing sequence generation and allocation concealment of the participant through FA.

Blinding was not feasible except for the statistician.

### Statistical analysis

Qualitative data were presented as frequencies and percentages. Chi-square test and Fisher’s Exact test were used for comparisons regarding qualitative data. Quantitative data were presented as mean and standard deviation (SD) values. Student’s t-test was used to compare between two groups. The significance level was set at P ≤ 0.05 for all tests. Statistical analysis was performed with IBM SPSS Statistics for Windows, Version 23.0. Armonk, NY: IBM Corp.

**Figure:**
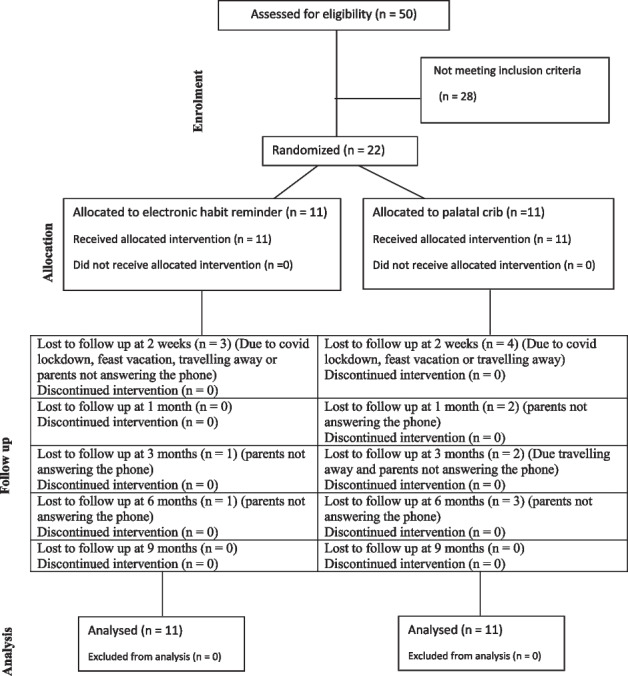
CONSORT diagram showing the flow of participants through each stage

## Results

### Demographic data

There was no statistically significant difference between mean age values in both groups with a mean ± SD 8.2 ± 1.7 and 8.3 ± 1.9 in the intervention and control group respectively. Also, there was no statistically significant difference between gender distributions in both groups.

### Comparison between Intervention and control groups

#### Cessation of sucking habit is shown in table 1

**Table 1:** Shows the descriptive statistics and results of fisher’s Exact test for comparison between cessation of sucking habit in both groups

Cessation	Intervention (*n* = 11)		Control (*n* = 11)		*P*-value
	n	%	n	%	
Yes	3	27.3	6	54.5	0.387
No	8	72.7	5	45.5	

#### Age, gender and cessation of sucking habit

There was no statistically significant difference between mean age values in both groups with a mean ± SD 8.5 ± 2.2 and 8.1 ± 1.5 in the children who ceased the habit and who did not cease the habit respectively. Also, there was no statistically significant difference between gender distributions in both groups regarding habit cessation.

#### Child acceptance

There was acceptance of treatment in both intervention and control groups 72.7% and 81.8% respectively. But there was no statistically significant difference between the acceptances of treatment in both groups.

#### Parental acceptance questionnaire regarding the intervention group is shown in table 2

**Table 2:** Descriptive statistics for parental acceptance questionnaire in intervention group (*n* = 11)

Questionnaire	Very		Somewhat		Neutral		Minimally		Not	
	n	%	n	%	n	%	n	%	n	%
1. Did you feel the device was successful in stopping habit?	5	45.5	3	27.3	1	9.1	1	9.1	1	9.1
2. Were instructions and information useful?	2	18.2	6	54.5	3	27.3	0	0	0	0
3. How easy was the device to use?	3	27.3	3	27.3	4	36.4	1	9.1	0	0
4. Would you recommend this technique?	4	36.4	4	36.4	1	9.1	1	9.1	1	9.1

#### Time for habit cessation (days/months) is shown in table 3

**Table 3:** Showing participants who ceased the thumb/finger sucking habit in the intervention and control groups at 6m and 9m

	Ceased at 6 m	Ceased at 9 m	Total
	(*n* = 6)	(*n* = 3)	
Intervention	1	2	3
Control	5	1	6

The original timeline for treatment was 6 months but it was extended to 9 months for actual effects to be recorded.

#### Finger used in sucking habit

Most of the cases used the thumb 72.7% and 90.9% in intervention and control group respectively. While there was only one case in both groups using pointer and only two cases using middle finger in the intervention group. But there was no statistically significant difference between fingers used in sucking habits in both groups. Also, there was no statistically significant difference between fingers used in sucking habits and those who ceased habit or not.

#### Problems with the appliances

The most common problem with the palatal crib appliance was dislodgement then re-cementation (63.6%). This was followed by fall more than once and band fracture (36.3%) for each problem, respectively. The least common problems were dislodgement without cementation and solder failure (18.1%) for each problem, respectively. While electronic habit reminder breakage more than once was 36.3%.

#### Breastfeeding, bottle-feeding and working mothers’ relation to thumb/finger sucking habit in the intervention and control group is shown in table 4

**Table 4:** Descriptive statistics and results of Fisher’s Exact test for comparison between breastfeeding, bottle-feeding and working mothers in both groups

	Intervention		Control		*P-value*
	*n* = 11		*n* = 11		
	n	%	n	%	
Breastfeeding					
yes	10	90.9	9	81.8	1
no	1	9.1	2	18.2	
Bottle-feeding					
yes	6	54.5	4	36.4	0.392
no	5	45.5	7	63.6	
Working mother					
yes	4	36.4	3	27.3	1
no	7	63.6	7	63.6	
other	0	0	1	9.1	

### Breastfeeding, bottle-feeding, working mothers and cessation of sucking habit is shown in table 5

**Table 5:** Descriptive statistics and results of Fisher’s Exact test for comparison between breastfeeding, bottle-feeding and working mothers among the children who ceased or did not cease the habit

	Ceased habit		Did not cease habit		*P-value*
	*n* = 9		*n* = 13		
	n	%	n	%	
Breastfeeding					
yes	6	66.7	13	100	0.055
no	3	33.3	0	0	
Bottle-feeding					
yes	5	55.6	5	38.5	0.666
no	4	44.4	8	61.5	
Working mother					
yes	0	0	7	53.8	0.017*
no	8	88.9	6	46.2	
other	1	11.1	0	0	

^*^Significant at *P* ≤ 0.05

##  Harms

Regarding the palatal crib appliance, there was gingival inflammation that resolved by proper oral hygiene care. Also, interference with speech which disappeared after adaptation.

Regarding appliances breakage or dislodgment, it was repaired or replaced with another one.

## Discussion

Oral habits are common among young children especially with age range 3 to 5 years old. These habits have different effects on growing children at variable levels psychologically and physically. One of these oral habits is thumb/finger sucking which starts as early as 29 weeks intrauterine in infants. Despite the early start of this habit but it is considered normal till the age of 5. Some studies recommend stoppage of the sucking habit by the age of 3 as it has different effects on occlusion that may extend to permanent dentition.

This study aimed to evaluate the electronic habit reminder to overcome the drawbacks of the palatal crib as it affects speaking, eating, and causes gingival inflammation. According to Dean, fixed palatal crib is commonly used and recommended for treating thumb/finger sucking habit. It was reported that 80% of children treated with fixed palatal crib stopped the thumb sucking habit in a week from the insertion of the appliance but they reported the need for at least 6 months for retention [22]. The electronic habit reminder in the form of a wristwatch was presented as a case report by Krishnappa et al. [18].

This study is considered a pilot study as the electronic habit reminder was only reported in one case report. The main purpose of a pilot study is to test acceptability and feasibility of a method to answer the question if this procedure can be applied or not similar to what was described by In [23]. Randomization was applied in this study to make sure equal distribution of patients among the two groups similar to Lim and In [24].

In the current study, cessation of sucking habit was higher in the control group with fixed palatal crib although it is not considered statistically significant. This agrees with Borrie et al*.*, who reported success of appliance treatment on the short and long term [25]. Also, Chhabra and Chhabra, who reported the use of a fixed appliance with positive reinforcement and motivation [26]. On the contrary, Casamassimo, reported in their study success of cessation in a large number of the participants used removable thumb guard due to their high compliance [19].

Among the two groups of participants in the current study, most of them accepted the treatment which agrees with the case report by Krishnappa et al. due to the attractive shape of the appliance and easiness to wear [18]. This agrees with Casamassimo, who reported that the parents said that the appliance was easy to wear despite complex attachment [19]. Child acceptance of the treatment is crucial, if it does not occur or the child feels that it’s a punishment. Therefore, treatment will fail or the habit will transfer to another form according to Moore [27].

Regarding the success of the appliance in the intervention group in our study, most of the parents found it successful which agrees with Casamassimo in patients using thumbguard [19]. Almost half of the parents reported the easiness of use similar to Casamassimo [19]. Most of the parents recommended the use of the appliance like the parents in Casamassimo [19]. Also, a large percentage of the parents in our study found the instructions and information provided were useful as the parents in Casamassimo study [19].

In this study, nearly half of the participants ceased the thumb/finger sucking habit by the end of the trial. Two third of them ceased the habit at 6 months which agrees with Krishnappa [18].

In our study, the mean age of the participant agreed with Casamassimo [19]. As most oral habits start by the first year and continue at a constant level till 7 years according to Bishara et al. [7]. Jajoo et al*.* reported that the prevalence of sucking habits in India was from age 5–7 years due to lack of care and love this was explained by two theories the psychosexual development (psychoanalytic theory) and the other one that sucking habit is adaptive behavior [28].

Omer and Abuaffan, found the prevalence of the habit increase with age with no statistical significance [29]. On the contrary, Dos Santos et al*.* & Varas et al. reported that the Brazilian and Spanish children’s sucking habit prevalence were more in younger age [30, 31]. Marwah, reported that the habit is present in neonates as expression of primitive demands as hunger, in the first weeks postpartum due to feeding problems while older than that due to teething [32].

Our study found nearly equal distribution of the habit between males/females that is not statistically significant, which is in disagreement with Zakirulla et al*.,* Thadchanamoorthy and Dayasiri & Maia-Nader et al*.* in Sri Lanka, South Asia and Brazil who reported that females were more prevalent in thumb/finger sucking habit [34–36].

In our study, we found that most of the participants where thumb sucking and the remaining where digit sucking which agrees with Farsi and Pedo who reported the same in Saudi children [33]. It may be attributed to the way they are raised, which differs between populations. Larsson et al*.* & Thadchanamoorthy and Dayasiri, reported that thumb sucking was more prevalent than digit sucking in the dominant hand, which is in agreement with our study [34, 38]. On the contrary, Ramesh et al., reported that children sucking habit using digit more than thumb as it is easy to access, and their findings were due to small sample size over large area [39].

In our study, we found that palatal crib appliance fall, and re-cementation was the most common problem which is three times what Chhabra and Chhabra reported in their study due to improper placement and distortion during initial insertion. This was followed by appliance breakage in agreement with Chhabra and Chhabra [26]. Few of the participants in our study, dislodged the appliance by themselves in the same day of cementation despite the instructions of not playing with appliance by the tongue or finger and not to eat for two hours like Moore who found that children who are out of control remove the appliance and it is recommended to re-cement it. While unwilling children to stop the habit may deform the appliance and break it [27]. In our study, dislodgement without cementation was low due to loss of contact with the parents, moving away or the parents were busy with work. Borrie et al*.*, stated that there was no available information about the fitting, cementation, breakage and removing the appliance [25].

In our study, most of the participants with thumb/finger sucking habit were breastfed and a very small percent were bottle fed but it was not statistically significant. This is in disagreement with Farsi and Pedo; Maia-Nader et al*.* & Omer and Abuaffan who reported that the lowest prevalence of thumb/finger sucking habit was among breastfed children, this may be attributed to the young age range of the participants and culture differences [33, 36, 29]. AlSadhan and Al-Jobair stated in their study, that there were high prevalence of digit sucking among bottle fed orphan children due to lack of awareness of foster mothers or due to imitation of other children [37].

Bottle feeding more than 18 months has no influence on increasing the prevalence of digit sucking habit. Early weaning from breastfeeding did not increase the prevalence of sucking habits and this is explained by the close contact during breastfeeding that satisfy their sucking needs according to Chen et al. [1]. In agreement with our findings, Ramesh et al. reported in their study that nearly twice the percentage of the participants who breastfeed for short period had malocclusion and NNS (Non-nutritive sucking habit) [39]. WHO recommends exclusive breastfeeding for the first 6 months [40].

In our study, most of the mothers were not employed in either group but it was statistically insignificant. In agreement to our findings Thadchanamoorthy and Dayasiri, in Sri Lanka found a high percentage of sucking habits in the children with housewives’ mothers. This is attributed to demographic, socioeconomic and racial conditions [34]. While, there are several studies disagree with our findings as Maia-Nader et al*.*, reported that Brazilian mothers working away from their children led to high prevalence of sucking habit in their children due to early cessation of breastfeeding causing frustration to the child [36]. Al-Hussyeen & Chopra et al*.* stated that the children of working mothers who are absent for a long time affects breastfeeding and sense of secureness adversely affects sucking habits [2, 41]. This is attributed to the fact that the caretaker bring up their children and they may develop sense of insecurity as mentioned by Marwah [32].

The strength of our study is the application of randomization and allocation concealment in the process of appliance selection for every patient. Additionally, the removable wristwatch has the advantage of being custom made for every patient and close fitting to the hand, it can be used for finger sucking not only for thumb like several appliances. The wristwatch can be changed according to the interest of the child to be more attractive and acceptable, like changing the colour and theme.

The limitation of this study is that it depends mainly on the compliance of the child on wearing the removable appliance all the time and the cooperation of the parent to encourage the child to stop the habit, being with the child to make sure he/she wears the appliance. Also, some parents refuse to make their child wear the appliance at school due to fear of its loss or breakage. Additionally, some children can remove the appliance when the parent is not around him/her or removes it during sleep. Some parents did not report the fall of the fixed appliance because they were busy which causes the child to relapse making the treatment period extend.

## Conclusions

Cessation of thumb/finger sucking habit in control group was higher. While Participant’s acceptance of treatment was high in both groups but compliance on the long term was low in the intervention group. Parental acceptance regarding success of treatment, ease of use of the appliance was high as they recommend other parents to use the same appliance. It is worth mentioning that there was no statistical significant difference between both groups in regard to cessation of the habit.

It is recommended to provide proper education of the parents about the effects of thumb/finger sucking habits on the child physically and psychologically. Knowledge about seeking paediatric dental assistance for cessation of thumb/finger sucking habit and different treatment modalities according to each age range. Further studies for the electronic habit reminder should be carried out with larger sample size, and development of a more advanced appliance that withstand long term use without breakage or deformation. Also, future studies exploring duration and frequency of thumb/ finger habits are needed.

## Data Availability

The data set used and/or analyzed during the study is available from the corresponding author upon reasonable request.
